# Differential Effects of HOXB4 on Nonhuman Primate Short- and Long-Term Repopulating Cells

**DOI:** 10.1371/journal.pmed.0030173

**Published:** 2006-05-02

**Authors:** Xiao-Bing Zhang, Brian C Beard, Katherine Beebe, Barry Storer, R. Keith Humphries, Hans-Peter Kiem

**Affiliations:** 1Clinical Research Division, Fred Hutchinson Cancer Research Center, Seattle, Washington, United States of America; **2**Department of Biostatistics, University of Washington, Seattle, Washington, United States of America; **3**Terry Fox Laboratory, British Columbia Cancer Agency, Vancouver, British Columbia, Canada; **4**Department of Medicine, University of British Columbia, Vancouver, British Columbia, Canada; **5**Departments of Medicine and Pathology, University of Washington, Seattle, Washington, United States of America; Erasmus UniversityNetherlands

## Abstract

**Background:**

Hematopoietic stem cells (HSCs) or repopulating cells are able to self-renew and differentiate into cells of all hematopoietic lineages, and they can be enriched using the CD34 cell surface marker. Because of this unique property, HSCs have been used for HSC transplantation and gene therapy applications. However, the inability to expand HSCs has been a significant limitation for clinical applications. Here we examine, in a clinically relevant nonhuman primate model, the ability of HOXB4 to expand HSCs to potentially overcome this limitation.

**Methods and Findings:**

Using a competitive repopulation assay, we directly compared in six animals engraftment of HOXB4GFP (HOXB4 green fluorescent protein) and control (yellow fluorescent protein [YFP])–transduced and expanded CD34 ^+^ cells. In three animals, cells were infused after a 3-d transduction culture, while in three other animals cells were infused after an additional 6–9 d of ex vivo expansion. We demonstrate that *HOXB4* overexpression resulted in superior engraftment in all animals. The most dramatic effect of HOXB4 was observed early after transplantation, resulting in an up to 56-fold higher engraftment compared to the control cells. At 6 mo after transplantation, the proportion of marker gene–expressing cells in peripheral blood was still up to 5-fold higher for HOXB4GFP compared to YFP-transduced cells.

**Conclusions:**

These data demonstrate that *HOXB4* overexpression in CD34 ^+^ cells has a dramatic effect on expansion and engraftment of short-term repopulating cells and a significant, but less pronounced, effect on long-term repopulating cells. These data should have important implications for the expansion and transplantation of HSCs, in particular for cord blood transplantations where often only suboptimal numbers of HSCs are available.

## Abstract 2

Overexpression of the stem-cell self-renewal gene *HOXB4* improves expansion and early engraftment of hematopoietic stem cells in competitive repopulation assays in primates.

## Introduction

Hematopoietic stem cells (HSCs) have the unique property of being able to self-renew and differentiate into cells of all hematopoietic lineages. Expansion of HSCs has many potential applications in clinical settings, including stem cell transplantation and stem cell gene therapy. Cord blood transplantation has been increasingly used for the treatment of hematopoietic disorders because of its rapid accessibility and a lower risk of graft-versus-host disease even in a human leukocyte antigen mismatched setting [ 1]. However, the limited number of cord blood cells available in one unit, particularly for adult patients, has resulted in delayed engraftment and an increased risk of infections after cord blood transplantation [ 2, 3]. Thus, cord blood expansion has been proposed to improve engraftment [ 4]. Expansion of HSCs may also improve current HSC gene therapy protocols, e.g., an increased number of gene–modified/corrected cells may improve engraftment after nonmyeloablative conditioning [ 5]. Ex vivo expansion may also allow for improved safety screening for gene-modified clones harboring unfavorable integration sites.

The different approaches to stem cell expansion can be divided into three categories. First, the use of cytokines, such as Flt-3 ligand (Flt3-L), stem cell factor (SCF), and thrombopoietin (TPO) [ 6– 11]; second, the use of stroma, such as bone marrow stromal cells and endothelial cells, to support long-term culture [ 12– 16]; and third, the use of the stem cell self-renewal genes, such as *HOXB4* and genes in the Notch and WNT signaling pathways. Constitutively active Notch1 has been shown to immortalize murine HSCs and to favor lymphoid lineage differentiation rather than myeloid lineage differentiation [ 17]. Growth-enhancing effects of Notch4 on human cord blood HSCs have also recently been reported [ 18]. Expression of modified β-catenin or treatment of cells with WNT3a protein can expand mouse HSCs more than 100-fold without leukemogenesis [ 19, 20]. However, no marked effect on human HSCs was observed. Overexpression of the human *HOXB4* gene has been shown to induce ex vivo expansion and self-renewal of murine long-term multi-lineage repopulating HSCs without compromising differentiation or homeostatic regulation of the HSC pool size [ 21]. More recently, a TAT-HOXB4 fusion protein has been developed as a reagent that is able to stimulate expansion of mouse HSCs [ 22] when added to liquid cultures. Furthermore, in vivo and ex vivo expansion of human cord blood CD34 ^+^ cells by *HOXB4* overexpression or by direct delivery of HOXB4 was observed, although not to the same extent as observed in mouse cells [ 23– 25].

Experimental preclinical studies to determine the effect of *HOXB4* overexpression on human hematopoietic repopulating cells can be performed only in an xenogeneic transplantation model such as the NOD/SCID (nonobese diabetic/severe combined immunodeficient) mouse model, which is generally limited by the relatively short life span of the recipients and the inability to support differentiation into all hematopoietic lineages. Thus, in the current study we wished to examine the effect of *HOXB4* overexpression in a clinically relevant large-animal transplantation model. We chose the nonhuman primate model because it is a well-established preclinical model for HSC gene transfer, expansion, and transplantation. Using a competitive repopulation assay, we studied the effect of HOXB4 on hematopoietic repopulation with cells that were infused directly after a 3-d transduction period or after an additional 6–9 d of ex vivo expansion.

## Methods

### Animals

Pig-tailed macaques ( Macaca nemestrina) were housed in the primate center at the University of Washington. Animals were handled in accordance with the guidelines set by the American Association for Accreditation of Laboratory Animal Care, and the protocols were approved by the Animal Care and Use Committee of the Fred Hutchinson Cancer Research Center and the University of Washington.

Macaques were treated with recombinant human (rhu) G-CSF (Amgen, Thousand Oaks, California, United States), 100 μg/kg daily given as subcutaneous injections for 5 d. On day 5, either bone marrow was harvested from the humeri and/or femora, or mobilized peripheral blood cells were collected by leukapheresis. In preparation for transplantation, all animals received myeloablative total-body irradiation. 24 h after transplantation, the animals were started on G-CSF at 100 μg/kg daily, given as intravenous injection, until the animals started to engraft. Standard supportive care, including blood-product transfusions, fluid and electrolyte management, and antibiotics, was administered as needed. Hematopoietic recovery was monitored by daily complete blood counts. Animals R01019 and T02266 were euthanized by the veterinary staff owing to poor condition. Animal R01019 was euthanized on day 17. The animal had not recovered well from the irradiation, became hypothermic, and developed ischemic kidney damage. T02266 was euthanized on day 47. Necropsy findings revealed histopathological changes consistent with radiation damage. In addition, the animal appeared to have a papovavirus-induced nephritis. Findings in both animals were consistent with transplantation/irradiation–associated toxicity.

### Retrovirus Preparation

Derivation of the gammaretroviral vector plasmid MSCV-HOXB4-ires-GFP (green fluorescent protein) from the original MSCV backbone provided by R. Hawley [ 26] has been described previously [ 21]. The control, MSCV-ires-YFP (yellow fluorescent protein) was generated by inserting the coding region of the eYFP reporter gene in place of the eGFP reporter gene of the previously described MSCV-ires-GFP vector [ 21]. The vectors were used to transiently transfect 293T-based Phoenix-RD114 packaging cells. The resulting virus-containing media were used to transduce Phoenix-gibbon ape leukemia virus (GALV) packaging cells as described earlier [ 27]. A helper virus-free high-titer clone was selected. Virus-containing medium was collected in DMEM supplemented with 10% fetal bovine serum (FBS) and 1% penicillin/streptomycin from monolayers of Phoenix-GALV producer cells after incubation for 12 h at 37 °C. This cell line produced virus at a titer of 1 × 10 ^5^ to 2 × 10 ^5^ CFU/ml when assayed with HT1080 cells. Fresh vector supernatant was passed through a 0.22-μm filter (Millipore, Bedford, Massachusetts, United States) and preserved at −80 °C for later transduction.

### Transduction of CD34 ^+^ Cells

CD34 cell enrichment was performed using the 12.8 IgM anti-CD34 antibody and MACS IgM microbeads (Miltenyi Biotec, Auburn, California, United States) according to the manufacturer's instructions and as described previously [ 28, 29]. The average purity of CD34 ^+^ cells in the enriched product was 64% (range, 45%–79%) as determined by flow cytometry. CD34 ^+^ cells were split into two equal fractions; one fraction was transduced with the HOXB4 green fluorescent protein (HOXB4GFP) vector and the other fraction with the YFP control vector. The use of two different markers, GFP versus YFP, for the two different culture conditions allows for the use of a competitive repopulation approach, i.e., following transduction/expansion, cells from both fractions are pooled and infused into the recipient after myeloablative irradiation. Cells from the two different culture conditions can then be easily detected in vivo by analyzing peripheral blood cells for the presence of GFP ^+^ and YFP ^+^ cells. CD34-enriched macaque cells were cultured in Iscove's modified Dulbecco's medium (IMDM)/10% FBS supplemented with 100 ng/ml rhuSCF, 100 ng/ml rhuFlt3-L, 100 ng/ml interleukin (IL)-3, 100 ng/ml IL-6, 100 ng/ml Thrombopoietin (TPO), and 100 ng/ml G-CSF for 2 d before transduction. For transduction, 2.5 × 10 ^5^ cells/ml were cultured with filtered vector supernatant at a multiplicity of infection of 0.3, supplemented with the same cytokine combination in flasks previously coated with the CH-296 fragment of fibronectin (Retronectin, Takara, Shiga, Japan) for 4 h. 24 h later, nonadherent cells were collected, spun down, and resuspended in fresh vector supernatant and cytokines and added back to the same fibronectin-coated flask. 4 h later, cells were harvested for transplantation, colony-forming cell (CFC) assay, or long-term liquid culture.

### Long-Term Ex Vivo Culture

HOXB4GFP- or YFP-transduced cells were maintained in the same culture media as used for transduction in tissue-culture-treated 24-well plates (Nunc, Rochester, New York, United States). Cultures were split every week at a ratio of 1:2 to 1:10 and the cell density adjusted to 2–5 × 10 ^5^/ml with fresh media and cytokines. Once a week, some cells were harvested for flow analysis to determine the percentages of HOXB4GFP ^+^ or YFP ^+^ cells and for CFC assays.

### Ex Vivo Expansion of Macaque CD34 ^+^ Cells for Transplantation

G-CSF–primed macaque bone marrow cells were purified as described previously. CD34 ^+^ cells were cultured in IMDM/10% FBS with addition of SCF, TPO, Flt3-L, and G-CSF, each at 100 ng/ml. After transduction with HOXB4GFP- or YFP-containing viral vectors, cells were cultured in CH296-coated flasks with the same culture medium and cytokines. After a further 6–9 d of culture, all the cells were harvested for transplantation.

### CFC Assays

CFC assays were performed in two-layer agarose in MEM with 20% FBS supplemented with 4 U/ml rhu erythropoietin (Amgen), and SCF, GM-CSF, G-CSF, IL-6, and IL-3 each at 100 ng/ml at 37 °C. Between days 12 and 14, colonies of more than 50 cells were counted.

### Taqman PCR Analysis

HOXB4GFP-Genomic DNA was extracted using the QIAamp DNA blood kit (Qiagen, Valencia, California, United States). The primers and conditions used for GFP/YFP and β-actin PCR have been described previously [ 30].

For every PCR analysis, negative controls included DNA from normal peripheral blood samples extracted with the same methodology and a reagent control. Serial dilutions of DNA (containing a single copy of an integrated vector per cell) into normal macaque peripheral blood DNA were used as positive controls for generating the control regression curve.

### Flow Cytometry

Flow-cytometric analysis was performed for GFP/YFP expression using a Vantage instrument and CellQuest software (Becton Dickinson, San Jose, California, United States). More than 20,000 events were analyzed for each sample. Non-transduced cells were used as a control for the gating of positive cells. For subset analysis, anti-CD14-PE, anti-CD3-APC-Cy7, anti-CD13-APC, and anti-CD20-PE were used. All antibodies were purchased from Becton Dickinson.

### Linear Amplification–Mediated Polymerase Chain Reaction

Integration-site analysis by linear amplification–mediated polymerase chain reaction (LAM-PCR) was performed with DNA isolated from FACS (fluorescence-activated cell sorter)–sorted HOXB4GFP ^+^ or YFP ^+^ peripheral blood cells. One hundred nanograms of DNA was used as template for LAM-PCR that was performed as described previously with modifications [ 31, 32]. Briefly, TasI was used to digest DNA after initially amplifying and creating double-stranded DNA. An enzyme-specific linker cassette was ligated into the TasI-restriction overhangs, and two additional rounds of PCR amplified the virus long-terminal repeat and genomic flanking regions. PCR products were visualized on spreadex gels (Elcrom Scientific, Cham, Switzerland).

### Western Blot

White blood cells of bone marrow or peripheral blood samples from animals were obtained by lysis buffer treatment. HOXB4GFP ^+^ cells were sorted by FACS to a purity of 99%. Proteins were extracted by radio-immunoprecipitation assay buffer with fresh addition of 1% protease-inhibitor cocktail (Sigma, St. Louis, Missouri, United States). Proteins were separated by SDS-polyacrylamide gel electrophoresis and subsequently blotted onto PVDF membranes. HOXB4 was detected after incubation with supernatant from I12, a hybridoma secreting rat anti-mouse HOXB4 antibody, which cross-reacts with human HOXB4 (I12; Developmental Studies Hybridoma Bank, University of Iowa, Iowa, United States). Visualization of bound antibodies was performed with the goat anti-rat horseradish peroxidase–conjugated secondary antibody with subsequent enhanced chemiluminescence reaction (ECL Kit, Amersham Biosciences, Little Chalfont, United Kingdom). The membranes were stained with Bluestain (Pierce Biotechnology, Rockford, Illinois, United States) as protein-loading controls.

### Statistical Analysis

Student's paired *t*-tests were used for the analysis of both in vitro and in vivo comparisons. For the in vivo analyses, we used days 1–50 to analyze early engraftment and days 51 and onwards to analyze late engraftment. Within each time period, the marking was averaged within each monkey. Paired differences were then computed for each monkey or culture experiment, either comparing HOXB4GFP and YPF marking within the same time period, or comparing HOXB4GFP marking between time periods. A log-transformation was applied for the analysis of fold increase in total cell expansion and CFC yield in culture before computing paired differences.

## Results

### HOXB4-Mediated Expansion of M. nemestrina Progenitor Cells

**Figure 1:**
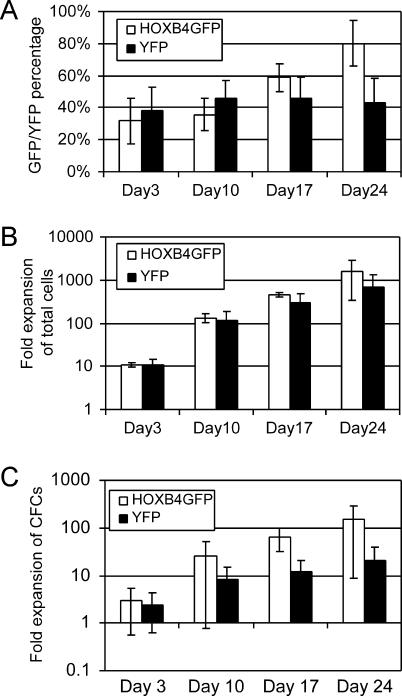
Efficient HOXB4-Mediated Ex Vivo Expansion of M. nemestrina CD34 ^+^ Cells The mean and standard deviation of four independent experiments are shown. HOXB4GFP- or YFP (control)–transduced M. nemestrina bone marrow CD34 ^+^ cells were kept in liquid culture for 4 wk. Culture media were changed regularly to keep cells at optimum densities. (A) Percentage of HOXB4GFP ^+^ or YFP ^+^ cells. (B) Fold expansion of total nucleated cells. (C) Fold expansion of CFCs.

We first investigated the effects of *HOXB4* overexpression on ex vivo expansion of CD34 ^+^ cells from macaque bone marrow. CD34 ^+^ cells were transduced with an HOXB4-expressing gammaretroviral vector MSCV-HOXB4-ires-GFP or with a control vector MSCV-ires-YFP. After culture, the percentage of *HOXB4-*overexpressing macaque cells (detected by expression of the linked GFP reporter gene) increased from 32.4% (standard deviation 12.2%) on day 3 to 81.0% (standard deviation 12.2%) on day 24, while the proportion of YFP ^+^ (control)–transduced cells remained essentially unchanged, from 38% (standard deviation 14.7%) on day 3 to 43.5% (standard deviation 15.4%) on day 24. A significant difference was observed on day 24 ( *p* = 0.007, paired *t*-test; *n* = 4). *HOXB4* overexpression was also associated with a 3- to 5-fold increase in total cell expansion (mean 1,720-fold, standard deviation 1,214-fold) on day 24 when compared with YFP-transduced control cells (mean 671-fold, standard deviation 637-fold) on day 24 ( *p* = 0.01, paired *t*-test) ( Figure 1). An even more pronounced effect of HOXB4 was observed on CFC progenitors. *HOXB4* overexpression induced an up to 7-fold increase in the expansion of CFCs (mean 147-fold, standard deviation 141-fold) compared to the YFP control (mean 21-fold, standard deviation 18-fold) after 24 d in in vitro culture ( *p* = 0.005, paired *t*-test). Taken together, *HOXB4* overexpression conferred a growth advantage to total nucleated cells and CFC progenitors in long-term liquid culture.

### Improved Engraftment of HOXB4GFP-Transduced Repopulating Cells

We further investigated whether HOXB4 would allow for expansion of repopulating HSCs. Mobilized CD34 ^+^ peripheral blood cells were divided into two equal fractions for transduction with the HOXB4GFP- or YFP-expressing gammaretroviral vector. As shown in Table 1, the expansion, the number of CFCs infused, and the transduction efficiency were similar between HOXB4GFP- and YFP-transduced cells. Immediately after the 3-day transduction period, all the cells were transplanted into myeloablated animals ( *n* = 3).

**Table 1:**
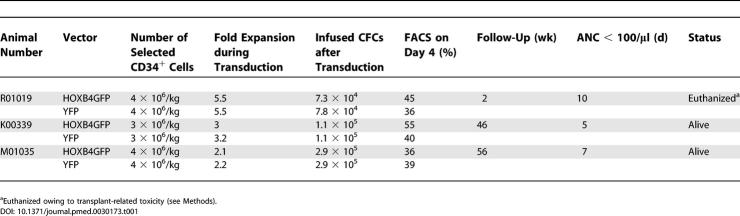
Summary of CD34 Enrichment, Infusion, Transduction Efficiency, and Follow-Up of the Animals Transplanted with HOXB4GFP- or YFP-Transduced Cells

**Figure 2:**
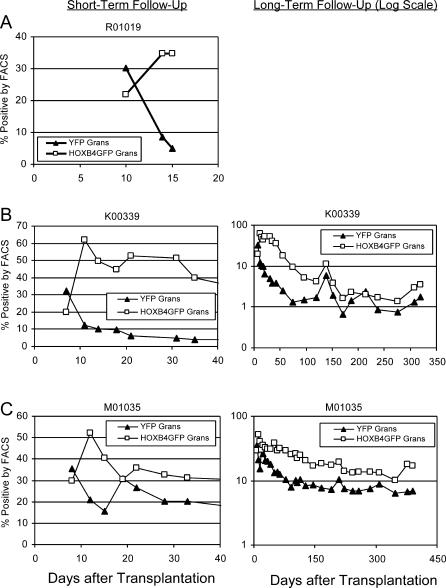
Improved Engraftment of HOXB4GFP-Transduced CD34 ^+^ Cells Equal numbers of macaque CD34 ^+^ cells were transduced in 3-d transduction cultures with either the HOXB4GFP- or YFP-expressing vector. Transduced cells were infused into myeloablated animals. The percentage of HOXB4GFP ^+^ and YFP ^+^ granulocytes was assessed by flow cytometry. Shown is the engraftment of HOXB4GFP ^+^ and YFP ^+^ granulocytes after transplantation.

In all three animals, R01019, K00339, and M01035, we observed a similar engraftment pattern for HOXB4GFP- and YFP-expressing cells ( Figure 2). Although the engraftment levels for HOXB4GFP ^+^ and YFP ^+^ cells were similar during the first 2 wk after transplantation, the percentage of HOXB4GFP-expressing cells increased dramatically thereafter while the percentage of YFP-expressing cells decreased in all animals. These engraftment kinetics led to a 2- to 9-fold higher percentage of HOXB4GFP-expressing cells than YFP-expressing cells between 2 and 7 wk after transplantation. Across the time period, the engraftment of HOXB4GFP-expressing cells (mean 36.9%, standard deviation 7.1%) was more than double that of YFP-expressing cells (mean 15.8%, standard deviation 6.1%) ( *p* = 0.08, paired *t*-test).

Marking levels eventually also declined for HOXB4GFP-marked cells before they stabilized. In M01035, HOXB4GFP marking stabilized at 15%–20% and YFP marking stabilized at 7%–10% at 6 mo post-transplantation, and marking levels remained stable for more than 1 y ( Figure 2). Marking levels in K00339 stabilized at 1%–2% for HOXB4GFP and 0.5%–1% for YFP. No immune responses were detected by lymphocyte-proliferation assays to explain the decline in gene-marked cells [ 33]. Gene-marking results as determined by Taqman PCR showed higher levels than the flow-cytometric data; however, the overall pattern was similar to the flow-cytometric results. Thus, the decline in GFP ^+^ and YFP ^+^ cells over time was not due to gene silencing, and most likely reflected the engraftment pattern of short-term repopulating cells.

Taken together, these results demonstrate a marked effect of *HOXB4* overexpression on engraftment of short-term repopulating cells with a less pronounced effect on long-term repopulating cells.

### Improved Engraftment of HOXB4GFP-Transduced and HOXB4GFP-Expanded Repopulating Cells

Based on the encouraging effect observed with HOXB4GFP-transduced cells transplanted immediately after transduction, we investigated whether more prolonged ex vivo culture after transduction would lead to further amplification of the HOXB4 effect. To that end, cells were cultured for an additional 6 d (K03290 and J02152) or 9 d (T02266) after the transduction culture. Cells were cultured in the presence of SCF, TPO, Flt3-L, and G-CSF. Table 2 shows the expansion and transduction efficiency of HOXB4GFP- and YFP-expanded cells before infusion into myeloablated animals.

**Table 2:**
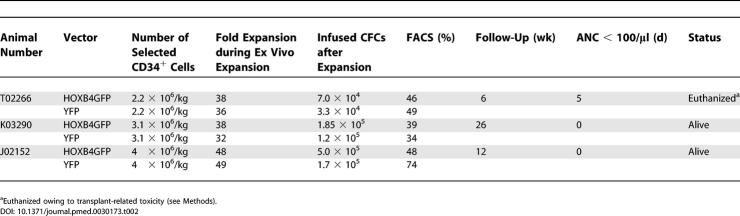
Summary of CD34 Enrichment, Ex Vivo Expansion of Total Cells and Infused CFCs, Transduction Efficiency, and Follow-Up of the Animals Transplanted with Ex Vivo Expanded HOXB4GFP- or YFP-Expressing Cells

**Figure 3:**
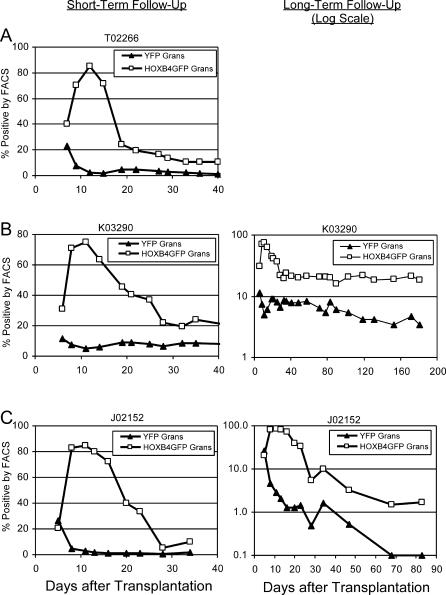
Improved Engraftment of HOXB4GFP-Transduced and HOXB4GFP-Expanded CD34 ^+^ Cells Equal numbers of macaque CD34 ^+^ cells were transduced in 3-d transduction cultures with either the HOXB4GFP or YFP vector and then cultured for an additional 9 d (T02266) or 6 d (K03290 and J02152) in the presence of SCF, TPO, Flt-3L, and G-CSF. All the transduced and expanded cells were infused into myeloablated animals. The percentage of HOXB4GFP ^+^ and YFP ^+^ granulocytes was assessed by flow cytometry. Shown is the engraftment of HOXB4GFP ^+^ and YFP ^+^ granulocytes after transplantation. (A) T02266, (B) K03290, and (C) J02152.

CD34-enriched cells from the first macaque (T02266) were cultured for an additional 9 d after transduction. As shown in Figure 3A, at 1 wk after transplantation, marking in granulocytes was significantly higher with the HOXB4GFP-expanded cells (up to 85%) than with control YFP ^+^ cells. 2 wk after transplantation, the difference was up to 35-fold, suggesting HOXB4-mediated ex vivo expansion of cells that contribute to early engraftment.

CD34-enriched cells from K03290 and J02152 were cultured for an additional 6 d before transplantation. A similar pattern in marking compared to T02266 was observed. In both K03290 and J02152, HOXB4GFP marking levels early after transplantation were between 75% and 85%. In K03290, the percentage of *HOXB4-*overexpressing cells was up to 14-fold higher than the percentage of YFP-expressing cells on day 11 after transplantation. 5 mo after transplantation, HOXB4GFP-marked granulocytes stabilized at 20% and YFP ^+^ cells stabilized at around 4%, representing a 5-fold difference ( Figure 3B). In J02152, the maximum difference between HOXB4GFP and YFP was 56-fold on day 16 ( Figure 3C). As shown in Table 2, in two out of the three animals that received HOXB4-expanded cells, the absolute neutrophil count (ANC) never dropped below 100/μl. Absolute numbers of gene-marked cells are shown in Figure S1.

HOXB4-mediated ex vivo expansion resulted in significantly higher engraftment of granulocytes early (<50 d) after transplantation (mean 38.1%, standard deviation 5.8%) compared to engraftment after growth-factor-only expansion (mean 5.7%, standard deviation 2.0%). ( *p* = 0.01, paired *t*-test). As with the animals that received HOXB4GFP-transduced CD34 ^+^ cells without any additional ex vivo expansion, the difference between HOXB4-mediated and growth-factor-only expansion was less pronounced after day 50. In the four animals that were assessed after day 50 (with and without ex vivo expansion), the HOXB4-mediated engraftment (mean 11.5%, standard deviation 9.5%) was only marginally higher than engraftment with YFP ^+^ cells (mean 3.8%, standard deviation 3.8%) ( *p* = 0.09, paired *t*-test). However, in these same four animals, the engraftment of HOXB4GFP ^+^ cells was significantly higher before day 50 (mean 40.7%, standard deviation 4.0%) compared to engraftment after day 50 (mean 11.5%, standard deviation 9.5%) ( *p* = 0.02, paired *t*-test).

### HOXB4-Mediated Expansion Results in Polyclonal Repopulation

**Figure 4:**
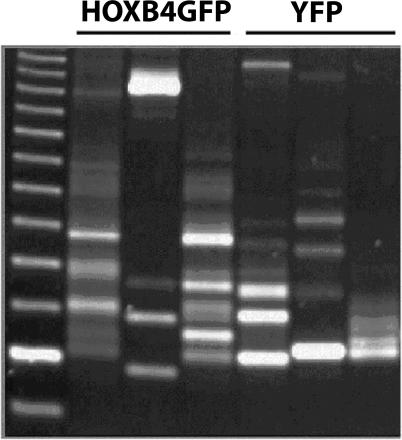
HOXB4-Mediated Expansion Results in Polyclonal Repopulation LAM-PCR was performed on lysates of sorted peripheral blood cells from K03290 at 3 mo post-transplantation. The lane on the far left is a 25-bp DNA ladder. HOXB4GFP indicates a triplicate LAM-PCR analysis for DNA from HOXB4GFP ^+^ cells. YFP indicates a triplicate LAM-PCR analysis for DNA from YFP ^+^ cells.

To determine whether *HOXB4* overexpression expanded multiple stem cell clones, we analyzed the clonal contribution of HOXB4GFP- or YFP-transduced cells to repopulation in K03290. As shown in Figure 4, integration-site analysis revealed multiple clones for both HOXB4GFP- and YFP-transduced cells. There was no obvious difference in the number of unique bands derived from HOXB4-overexpressing or YFP-expressing cells, suggesting that HOXB4-mediated stem cell expansion in this setting allowed for polyclonal stem cell expansion.

### Effects of *HOXB4* Overexpression on Hematopoietic Subpopulations

**Figure 5:**
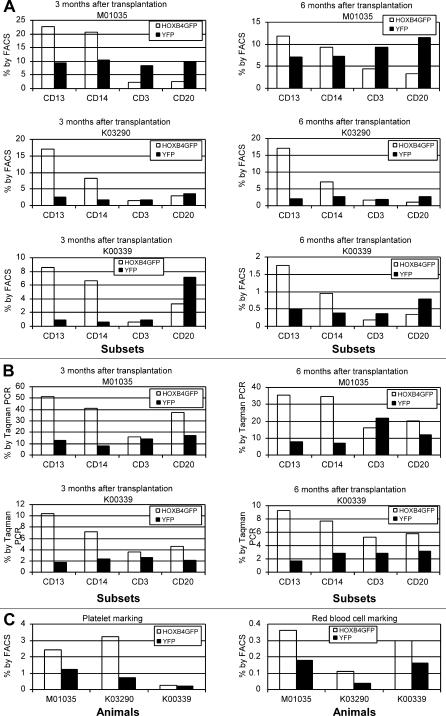
Differential Effects of *HOXB4* Overexpression on Granulocyte and Lymphocyte Engraftment and Marking Levels in Platelets and Red Blood Cells (A) Flow-cytometric analysis of CD13 ^+^, CD14 ^+^, CD3 ^+^, and CD20 ^+^ cells in peripheral blood of M01035, K00339, and K03290 at 3 and 6 mo after transplantation. (B) HOXB4GFP or YFP marking levels of FACS-sorted CD13 ^+^ myeloid cells, CD14 ^+^ monocytes, CD3 ^+^ T cells, and CD20 ^+^ B cells from peripheral blood of M01035 and K00339 at 3 or 6 mo after transplantation as determined by Taqman PCR. (C) Representative flow-cytometric analysis of marking levels in platelets and red blood cells at 3 mo after transplantation for M01035, K00339, and K03290.

At 3 and 6 mo post-transplantation, HOXB4GFP ^+^ or YFP ^+^ cells were present in CD13 ^+^ granulocytes, CD14 ^+^ monocytes, CD3 ^+^ T cells, and CD20 ^+^ B cells ( Figures 5A and S2). Flow-cytometric analysis showed that the percentage of HOXB4GFP ^+^ cells was higher in CD13 ^+^ and CD14 ^+^ cells as compared to YFP ^+^ cells, and in CD3 ^+^ and CD20 ^+^ cells this ratio was reversed.

Differential silencing of HOXB4GFP in lymphocytes versus granulocytes could have contributed to the differences in HOXB4GFP ^+^ cells in these subpopulations. To address this issue, we used Taqman PCR to measure the copies of HOXB4GFP or YFP integrants in FACS-sorted peripheral blood cells. As shown in Figures 5B and S3, the higher percentage of HOXB4GFP-expressing cells than YFP control cells in the myeloid fraction was confirmed. However, the difference identified by Taqman PCR between HOXB4GFP ^+^ cells and YFP ^+^ cells was less striking in the lymphoid fraction compared to the flow-cytometric results, suggesting some gene silencing or downregulation of HOXB4GFP in lymphocytes.

We also analyzed HOXB4GFP and YFP expression in red blood cells and platelets. Figure 5C shows a representative analysis of three animals at 3 mo after transplantation and demonstrates that more red blood cells and platelets are derived from HOXB4GFP ^+^- than from YFP ^+^-transduced cells. These data suggest that HOXB4 has no negative effect on the differentiation of megakaryocytes and erythrocytes.

Taken together, *HOXB4* overexpression appears to have a more pronounced effect on engraftment and differentiation of myeloid than T-lymphoid precursors.

### HOXB4 Protein Expression after Transplantation

**Figure 6:**
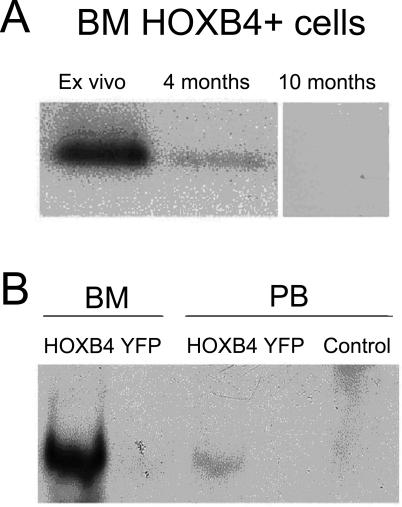
HOXB4 Protein Expression in HOXB4-Marked Cells (A) HOXB4-transduced CD34 ^+^ cells (lane 1) or FACS-sorted HOXB4GFP ^+^ bone marrow cells from M01035 at 4 mo (lane 2) or 10 mo post-transplantation (lane 3) were subjected to Western blot for detection of HOXB4 expression levels. (B) HOXB4GFP ^+^ or YFP ^+^ cells from bone marrow or peripheral blood in M01035 were isolated and subjected to Western blot analysis. Protein lysate of peripheral blood cells from a normal control animal was used as a negative control.

We observed a differential effect of *HOXB4* overexpression on short- and long-term repopulating cells. To investigate a putative mechanism, we examined HOXB4 expression in HOXB4GFP-transduced cells from animals after transplantation. At 4 and 10 mo after transplantation, HOXB4GFP-marked bone marrow cells were sorted by FACS to determine HOXB4 expression. While HOXB4 could be detected at 4 mo in bone marrow–derived CD34 ^+^ cells, no HOXB4 was detected at 10 mo after transplantation ( Figure 6A).

We also compared HOXB4 expression in sorted *HOXB4GFP-*overexpressing or YFP-expressing bone marrow or peripheral blood cells. No detectable HOXB4 expression was observed in YFP ^+^ bone marrow cells, confirming the specificity of the HOXB4 antibody. HOXB4 expression in HOXB4GFP-sorted bone marrow and peripheral blood cells was detected with a substantially higher level in the bone marrow at 4 mo after transplantation ( Figure 6B).

These results indicate differences in HOXB4 expression in bone marrow earlier and later following transplantation and also between bone marrow and peripheral blood. Thus, the less-pronounced effect on long-term repopulating cells may be due to downregulation of HOXB4 expression.

## Discussion

This is, to our knowledge, the first report evaluating HOXB4-mediated HSC expansion in a clinically relevant nonhuman primate model. We observed that *HOXB4* overexpression resulted in a dramatic increase in short-term repopulating cells compared to control-transduced cells. The effect was most pronounced when HOXB4GFP-transduced cells were expanded for an additional 6 to 9 d after transduction. Short-term engraftment was up to 56-fold higher with *HOXB4-*overexpressing cells than with control-transduced cells, potentially eliminating severe neutropenia. Even though the percentage of HOXB4GFP ^+^ cells declined over time, at 6 mo after transplantation the percentage of *HOXB4-*overexpressing cells was still up to 5-fold higher than the control, suggesting that *HOXB4* overexpression has an effect also on long-term repopulation.

In the three animals that received HOXB4GFP- or YFP-transduced cells after a 3-d transduction culture without further expansion, HOXB4GFP marking was lower than YFP marking during the first week after transplantation. This was independent of the transduction efficiency of each vector. In contrast, in the three animals transplanted with cells transduced and expanded ex vivo for 6–9 d, HOXB4GFP marking was higher than the YFP control at 1 wk after transplantation. These findings suggest that the in vivo effect observed in these animals was due to the expansion of short-term repopulating cells, which was more pronounced after an additional 6–9 d of expansion culture. This interpretation is also supported by the increased number of CFCs after the additional 6–9 d of culture. We observed greater differences in both short-term and long-term repopulating cells for the second group of animals transplanted with cells transduced and expanded ex vivo for 6–9 d compared with the first group; this may be due to an ex vivo growth advantage or to selection of *HOXB4*-overexpressing cells relative to the YFP control cells.

In the two animals (M01035 and K00339) that were followed for 56 and 46 wk, respectively, HOXB4GFP marking has remained 1- to 2-fold higher than YFP marking. For the three animals (T02266, K03290, and J02152) that received ex vivo-expanded cells, HOXB4GFP marking has remained about 3- to 5-fold higher than YFP marking after 5 wk post-transplantation. In the surviving animals (K03290 and J02152), this difference was maintained for 26 wk. These data suggest that *HOXB4* overexpression may also lead to expansion of long-term repopulating cells; however, under the culture conditions so far examined, the effect was less pronounced than the effect on early repopulating cells. Integration-site analyses also confirmed that HOXB4-mediated stem cell expansion led to a polyclonal repopulation.

*HOXB4* is the only gene known so far whose ectopic expression confers a selective growth advantage and a competitive repopulation advantage in vivo to murine HSCs, without compromising differentiation or homeostatic regulation of the HSC pool size [ 21]. No leukemogenesis has been reported by the infusion of *HOXB4*-transduced repopulating cells in mice. We also did not observe any leukemias or clonal abnormalities in our monkeys. We are closely monitoring by LAM-PCR the surviving animals for any development of monoclonality or leukemia. These data should provide us with unique information in a large-animal model with regard to the leukemogenic potential of HOXB4 and its potential effect on genes within close range of the HOXB4 insertion. The lack of any development of leukemia or monoclonality thus far may be in part explained by our finding that HOXB4 expression levels in vivo were lower than those in ex vivo culture, and that no HOXB4 expression could be detected in mature cells in peripheral blood. In addition, expression in bone marrow cells decreased over time after transplantation. These data suggest downregulation of HOXB4 expression after transplantation. The decrease in HOXB4 expression after transplantation may also be due to differential expression in progenitors and long-term repopulating cells.

HOXB4 promotes stem cell self-renewal, which may result in inhibition of differentiation or a delay in differentiation. HOXB4 expression-level-dependent inhibition of lymphomyeloid differentiation of human cord blood cells has been reported in the NOD/SCID mouse model [ 24]. We observed HOXB4GFP-marked cells in all hematopoietic subpopulations, but the difference between HOXB4GFP and YFP was greatest in granulocytes. In CD3 ^+^ T lymphocytes, the percentage of YFP ^+^ cells was higher than the percentage of HOXB4GFP ^+^ cells. These findings suggest preferential expansion of myeloid cells. The difference between myeloid and lymphoid expansion, however, was less pronounced compared to reports by Schiedlmeier et al. [ 24], emphasizing the importance of HOXB4 expression levels on lymphocyte and myeloid differentiation. In addition, our Taqman PCR results suggest that some of the decreased HOXB4GFP expression in lymphocytes may be due to decreased expression in these cells rather than to a block in lymphoid differentiation.

Our findings in the nonhuman primate model differ from results in the murine models, where HOXB4 overexpression appears to equally expand short- and long-term repopulating cells ([ 21]; unpublished data). This difference may be due to inherent differences in the biology of stem cells between mice and nonhuman primates. Another possible explanation is that HOXB4 expression in mouse cells is higher and potentially more stable ([ 24, 34]; H.-P. Kiem, unpublished data).

In conclusion, our results demonstrate that HOXB4-mediated expansion of CD34 ^+^ cells leads to a significantly improved engraftment of short-term repopulation cells with the potential to prevent severe neutropenia after myeloablative HSC transplantation. Our data also suggest that HOXB4 may provide a growth advantage to long-term repopulating cells. Thus, these data support further exploration of the benefits of HOXB4-mediated ex vivo expansion of stem cells to accelerate engraftment, particularly in the setting of cord blood transplantation where delayed engraftment is a significant concern and is associated with increased complications after transplantation [ 2, 3].

## Supporting Information

Figure S1 Kinetics of Absolute Numbers of HOXB4GFP- or YFP-Expressing Neutrophils in Three Animals that Received HOXB4GFP-Transduced and HOXB4GFP-Expanded Cells These data-points are calculated by multiplying the ANCs per μl by the percentages of HOXB4GFP- or YFP-expressing neutrophils.
(118 KB TIF)

Figure S2 Absolute Numbers of HOXB4GFP- or YFP-Expressing Myeloid and Lymphoid Cells as Detected by FACS for Three Animals at 3 or 6 mo after Transplantation HOXB4GFP ^+^ or YFP ^+^ cells in CD13 ^+^ myeloid cells, CD14 ^+^ monocytes, CD3 ^+^ T cells, and CD20 ^+^ B cells from peripheral blood of M01035, K00339, and K03290 were determined by FACS. Absolute numbers per μl of these subsets are calculated by multiplying the white blood cell count by the percentage of HOXB4GFP- or YFP- expressing cells within these subpopulations in peripheral blood.
(140 KB TIF)

Figure S3 Absolute Numbers of HOXB4GFP- or YFP-Expressing Myeloid and Lymphoid Cells as Detected by Taqman PCR for Two Animals at 3 or 6 mo after Transplantation CD13 ^+^ myeloid cells, CD14 ^+^ monocytes, CD3 ^+^ T cells, and CD20 ^+^ B cells from peripheral blood of M01035 and K00339 were sorted by FACS. DNA lysates of these samples were subjected to Taqman PCR analysis for the detection of HOXB4GFP- or YFP-integration events. Absolute numbers per μl of these subsets are calculated by multiplying the white blood cell count by the percentage of HOXB4GFP- or YFP- expressing cells within these subpopulations in peripheral blood.
(86 KB TIF)

**Patient Summary**

**Background.**

Stem cells of the blood system (also called hematopoietic stem cells [HSCs]) can make all the different cells found in our blood, including immune cells that protect us against diseases and platelets that stop bleeding after an injury. Only a very small number of blood cells are HSCs. A slightly higher proportion of the cells in the bone marrow are HSCs, and there is also a relatively high proportion of HSCs in the blood of the umbilical cord (which connects a baby to the mother during pregnancy). Doctors use HSCs to treat patients with genetic disorders of the blood or immune systems as well as those who have blood cancer (leukemia, lymphoma, or myeloma), because those cancers are treated with chemotherapy or radiation, which kills most blood cells. Stem cell transplantation can have serious side effects. These are less likely when the HSCs are as similar as possible to the patient's own blood cells. Finding a “good match” as a source for HSCs and then getting enough of the cells from that source for transplantation are important components of good treatment.

**Why Was This Study Done?**

Bone marrow has the highest concentration of HSCs. Until recently, it has been the primary source of HSCs for transplantation. As scientists are learning more about HSCs, the hope is that the smaller numbers of HSCs that are found in cord blood or regular blood can somehow be enriched or expanded, thus providing doctors with a much larger pool from which to choose well-matched HSCs. This is a study that tests one possible way of expanding HSCs, by inserting an active copy of a gene that gets stem cells to divide and make more stem cells. Insertion of the gene in question, called *HOXB4,* had shown promise in mouse experiments. The next step after rodent experiments, and before introducing an experimental new treatment to a small number of human patients in a clinical trial, is often to test the treatment in larger animals which more closely resemble human patients. For HSC transplantation, macaques (a type of monkey) are often used at this stage.

**What Did the Researchers Do and Find?**

They treated a total of six macaques in the following way: first they collected HSCs (mixed with some other blood cells) from each of the animals (some from bone marrow, and some from regular blood). They then divided the cells from each of the animals in half. Into one half, they introduced the *HOXB4* gene, together with a marker that makes the cells fluoresce in green. They treated the other half exactly the same, except that the only gene introduced was a marker that makes the cells fluoresce in yellow. They then grew the cells for a few days in the laboratory to expand them. After that, the monkeys received high-level radiation that killed off most of their remaining blood cells (which is similar to the aggressive treatment of blood cancer in a human patient). After such radiation, the animals die if they do not get an HSC transplant. The researchers next combined the two batches of cells from each animal and transplanted them back into the monkeys. They then followed the monkeys' recovery and checked what proportion of the transplanted cells that had moved to and settled in the bone marrow (i.e., had properly “engrafted”) contained the green or the yellow marker. They found clear differences between the “green” cells and the “yellow” cells. Particularly early after transplantation, there was a much higher proportion of green cells among the engrafted cells.

**What Do These Findings Mean?**

This suggests that HOXB4 might help to expand HSCs and speed up the process of engraftment. Given that patients with rapid engraftment have fewer complications, these results are encouraging. However, this was a small study, and additional studies are necessary before the decision can be made as to whether this treatment appears to be safe and effective and should be tested in human patients. Regarding future studies, the authors point out that HOXB4 is available as a recombinant protein (similar to other drugs like insulin). It may therefore be possible to expand HSCs directly with HOXB4 protein (as opposed to introducing the HOXB4 gene), thus avoiding any potential side effects from genetic manipulation of the cells.

**Where Can I Get More Information Online?**

Here are several Web sites with information on HSCs and transplantation of HSCs from different sources.

Fact sheet on bone marrow transplantation from the US National Cancer Institute: http://www.cancer.gov/cancertopics/factsheet/Therapy/bone-marrow-transplant

Pages from the American Cancer Society:

http://www.cancer.org (search within the site for stem cells, cord blood, and bone marrow transplantation)

Pages from the US National Cord Blood Program:

http://www.nationalcordbloodprogram.org

The US National Marrow Donor Program:

http://www.marrow.org

## Abbreviations

ANC: absolute neutrophil count
CFC: colony-forming cell
FACS: fluorescence-activated cell sorter
FBS: fetal bovine serum
Flt3-L: Flt-3 ligand
GALV: gibbon ape leukemia virus
GFP: green fluorescent protein
HOXB4GFP: HOXB4 green fluorescent protein
HSC: hematopoietic stem cell
IL: interleukin
IMDM: Iscove's modified Dulbecco's medium
LAM-PCR: linear amplification–mediated polymerase chain reaction
NOD/SCID: nonobese diabetic/severe combined immunodeficient
rhu: recombinant human
SCF: stem cell factor
TPO: thrombopoietin
YFP: yellow fluorescent protein

## References

[pmed-0030173-b001] Ballen KK (2005). New trends in umbilical cord blood transplantation. Blood.

[pmed-0030173-b002] Rocha V, Labopin M, Sanz G, Arcese W, Schwerdtfeger R Acute Leukemia Working Party of European Blood and Marrow Transplant Group, Eurocord-Netcord Registry (2004) Transplants of umbilical-cord blood or bone marrow from unrelated donors in adults with acute leukemia. N Engl J Med.

[pmed-0030173-b003] Laughlin MJ, Eapen M, Rubinstein P, Wagner JE, Zhang MJ (2004). Outcomes after transplantation of cord blood or bone marrow from unrelated donors in adults with leukemia. N Engl J Med.

[pmed-0030173-b004] McNiece I (2004). Ex vivo expansion of hematopoietic cells. Exp Hematol.

[pmed-0030173-b005] Neff T, Horn PA, Peterson LJ, Thomasson BM, Thompson J (2003). Methylguanine methyltransferase-mediated in vivo selection and chemoprotection of allogeneic stem cells in a large-animal model. J Clin Invest.

[pmed-0030173-b006] Zandstra PW, Conneally E, Petzer AL, Piret JM, Eaves CJ (1997). Cytokine manipulation of primitive human hematopoietic cell self-renewal. Proc Natl Acad Sci USA.

[pmed-0030173-b007] Zandstra PW, Lauffenburger DA, Eaves CJ (2000). A ligand-receptor signaling threshold model of stem cell differentiation control: A biologically conserved mechanism applicable to hematopoiesis. Blood.

[pmed-0030173-b008] Bhatia M, Bonnet D, Wu D, Murdoch B, Wrana J (1999). Bone morphogenetic proteins regulate the developmental program of human hematopoietic stem cells. J Exp Med.

[pmed-0030173-b009] Bhatia M, Bonnet D, Kapp U, Wang JC, Murdoch B (1997). Quantitative analysis reveals expansion of human hematopoietic repopulating cells after short-term ex vivo culture. J Exp Med.

[pmed-0030173-b010] Dorrell C, Gan OI, Pereira DS, Hawley RG, Dick JE (2000). Expansion of human cord blood CD34(+)CD38(-) cells in ex vivo culture during retroviral transduction without a corresponding increase in SCID repopulating cell (SRC) frequency: Dissociation of SRC phenotype and function. Blood.

[pmed-0030173-b011] Gupta P, Oegema TRJ, Brazil JJ, Dudek AZ, Slungaard A (2000). Human LTC-IC can be maintained for at least 5 weeks in vitro when interleukin-3 and a single chemokine are combined with O-sulfated heparan sulfates: Requirement for optimal binding interactions of heparan sulfate with early-acting cytokines and matrix proteins. Blood.

[pmed-0030173-b012] Breems DA, Blokland EA, Siebel KE, Mayen AE, Engels LJ (1998). Stroma-contact prevents loss of hematopoietic stem cell quality during ex vivo expansion of CD34+ mobilized peripheral blood stem cells. Blood.

[pmed-0030173-b013] Chute JP, Saini AA, Chute DJ, Wells MR, Clark WB (2002). Ex vivo culture with human brain endothelial cells increases the SCID-repopulating capacity of adult human bone marrow. Blood.

[pmed-0030173-b014] Kawano Y, Kobune M, Yamaguchi M, Nakamura K, Ito Y (2003). Ex vivo expansion of human umbilical cord hematopoietic progenitor cells using a coculture system with human telomerase catalytic subunit (hTERT)-transfected human stromal cells. Blood.

[pmed-0030173-b015] Kusadasi N, Koevoet JL, van Soest PL, Ploemacher RE (2001). Stromal support augments extended long-term ex vivo expansion of hemopoietic progenitor cells. Leukemia.

[pmed-0030173-b016] Torok-Storb B, Iwata M, Graf L, Gianotti J, Horton H (1999). Dissecting the marrow microenvironment. Ann N Y Acad Sci.

[pmed-0030173-b017] Varnum-Finney B, Xu L, Brashem-Stein C, Nourigat C, Flowers D (2000). Pluripotent, cytokine-dependent, hematopoietic stem cells are immortalized by constitutive Notch 1 signaling. Nat Med.

[pmed-0030173-b018] Vercauteren SM, Sutherland HJ (2004). Constitutively active Notch4 promotes early human hematopoietic progenitor cell maintenance while inhibiting differentiation and causes lymphoid abnormalities in vivo. Blood.

[pmed-0030173-b019] Reya T, Duncan AW, Ailles L, Domen J, Scherer DC (2003). A role for Wnt signalling in self-renewal of haematopoietic stem cells. Nature.

[pmed-0030173-b020] Willert K, Brown JD, Danenberg E, Duncan AW, Weissman IL (2003). Wnt proteins are lipid-modified and can act as stem cell growth factors. Nature.

[pmed-0030173-b021] Antonchuk J, Sauvageau G, Humphries RK (2002). HOXB4-induced expansion of adult hematopoietic stem cells ex vivo. Cell.

[pmed-0030173-b022] Krosl J, Austin P, Beslu N, Kroon E, Humphries RK (2003). In vitro expansion of hematopoietic stem cells by recombinant TAT-HOXB4 protein. Nat Med.

[pmed-0030173-b023] Buske C, Feuring-Buske M, Abramovich C, Spiekermann K, Eaves CJ (2002). Deregulated expression of HOXB4 enhances the primitive growth activity of human hematopoietic cells. Blood.

[pmed-0030173-b024] Schiedlmeier B, Klump H, Will E, Arman-Kalcek G, Li Z (2003). High-level ectopic HOXB4 expression confers a profound in vivo competitive growth advantage on human cord blood CD34+ cells, but impairs lymphomyeloid differentiation. Blood.

[pmed-0030173-b025] Amsellem S, Pflumio F, Bardinet D, Izac B, Charneau P (2003). Ex vivo expansion of human hematopoietic stem cells by direct delivery of the HOXB4 homeoprotein. Nat Med.

[pmed-0030173-b026] Hawley RG, Lieu FH, Fong AZ, Hawley TS (1994). Versatile retroviral vectors for potential use in gene therapy. Gene Ther.

[pmed-0030173-b027] Neff T, Peterson LJ, Morris JC, Thompson J, Zhang X (2004). Efficient gene transfer to hematopoietic repopulating cells using concentrated RD114-pseudotype vectors produced by human packaging cells. Mol Ther.

[pmed-0030173-b028] Kiem HP, Andrews RG, Morris J, Peterson L, Heyward S (1998). Improved gene transfer into baboon marrow repopulating cells using recombinant human fibronectin fragment CH-296 in combination with interleukin-6, stem cell factor, FLT-3 ligand, and megakaryocyte growth and development factor. Blood.

[pmed-0030173-b029] Horn PA, Topp MS, Morris JC, Riddell SR, Kiem HP (2002). Highly efficient gene transfer into baboon marrow repopulating cells using GALV-pseudotype oncoretroviral vectors produced by human packaging cells. Blood.

[pmed-0030173-b030] Kurre P, Morris J, Thomasson B, Kohn DB, Kiem HP (2003). Scaffold attachment region-containing retrovirus vectors improve long-term proviral expression after transplantation of GFP-modified CD34
^+^ baboon repopulating cells. Blood.

[pmed-0030173-b031] Neff T, Beard BC, Peterson LJ, Anandakumar P, Thompson J (2005). Polyclonal chemoprotection against temozolomide in a large-animal model of drug resistance gene therapy. Blood.

[pmed-0030173-b032] Schmidt M, Carbonaro DA, Speckmann C, Wissler M, Bohnsack J (2003). Clonality analysis after retroviral-mediated gene transfer to CD34(+) cells from the cord blood of ADA-deficient SCID neonates. Nat Med.

[pmed-0030173-b033] Morris JC, Conerly M, Thomasson B, Storek J, Riddell SR (2004). Induction of cytotoxic T-lymphocyte responses to enhanced green and yellow fluorescent proteins after myeloablative conditioning. Blood.

[pmed-0030173-b034] Beslu N, Krosl J, Laurin M, Mayotte N, Humphries KR (2004). Molecular interactions involved in HOXB4-induced activation of HSC self-renewal. Blood.

